# Evaluation d´une formation en sécurité transfusionnelle auprès des étudiants en médecine au Maroc

**DOI:** 10.11604/pamj.2024.47.84.35703

**Published:** 2024-02-26

**Authors:** Ouadii Abakarim, Fatima Ezzahra Lahlimi, Illias Tazi

**Affiliations:** 1 Service d´Hématologie et Greffe de Moelle Osseuse, Centre Hospitalier Universitaire Mohammed VI, Faculté de Médecine et de Pharmacie, Université Cadi Ayyad, Marrakech, Maroc

**Keywords:** Formation médicale, sécurité transfusionnelle, soins infirmiers, étudiants en médecine, Medical education, transfusion safety, nursing, medical students

## Abstract

**Introduction:**

la formation médicale au sein des facultés de médecine et des hôpitaux du monde entier abordent peu le sujet de la médecine transfusionnelle et nécessitent un renforcement des programmes universitaires actuels. Les indications transfusionnelles sont plus fréquentes que ce qui est recommandé, ce qui contribue à augmenter les risques liés à cette procédure. Pour pallier à ceci, une formation par objectif structuré a été organisée en gestes et soins infirmiers sous le thème de la sécurité transfusionnelle. Le but de notre étude était d´évaluer l´appréciation de cette formation par les étudiants en médecine et le degré de sa maîtrise auprès de cette population.

**Méthodes:**

il s´agit d´une étude transversale, descriptive et monocentrique. Nous avons effectué une enquête auprès des étudiants en 3^e^ année de médecine. Un questionnaire d´appréciation auto administré, ainsi qu´une grille d´évaluation des compétences acquises, rempli par les médecins formateurs au cours de la séance. L´analyse des données a porté sur la statistique descriptive à l´aide du logiciel.

**Résultats:**

trois-cent-quatre-vingt-quatre (n=384) étudiants ont été convoqués à cette formation dont 275 (71,6%) ont participé à l´étude. Le taux de satisfaction globale était de 93,8%. Les objectifs et l´organisation étaient concluants à 95,6%. La qualité de la formation était satisfaisante à 90,3%. Le choix du thème de la station était convenant à 80%, la fluidité de la séance à 86,3%, la qualité de l´organisation et du débriefing à 89%, l´interaction entre formateurs et apprenants à 90,2%, la motivation des formateurs et la réflexion suscitée chez les apprenants à 92%. Nous avons noté que 93% des étudiants n´ont jamais participé à une formation sur la sécurité transfusionnelle. La maîtrise des compétences globales était totale à 67%, partielle à 26% et absente à 7%. Les étudiants ont maitrisé les mesures de vérifications de l´identité et du groupage du produit à transfuser à 97%, le principe de l´interprétation du contrôle ultime pré transfusionnel à 96%, le but du contrôle ultime au lit du malade à 93% avec maîtrise de sa réalisation technique à 83%.

**Conclusion:**

la formation en sécurité transfusionnelle a reçu un accueil favorable avec une maîtrise satisfaisante. Cette expérience peut être facilement étendue à d´autres thèmes.

## Abstract (en)

**Introduction:**

students worldwide receive little training on blood transfusion therapies during their time at medical schools and hospitals, then reformulation of academic programs is mandatory. Indications for blood transfusions are more frequent than recommended, which helps to increase the risks associated with this procedure. To overcome this, structured objective training on nursing care and procedures was organised with the topic of transfusion safety. The purpose of our study was to assess medical students´ appreciation of this training and the degree to which this population had mastered it.

**Methods:**

we conducted a cross-sectional, descriptive, single-centre study. We conducted a survey among the 3^rd^-year medical students. A self-administered assessment questionnaire, as well as an evaluation grid for acquired skills, filled in by the training doctors during the session, were used. Data analysis was based on descriptive statistics using Excel software.

**Results:**

three hundred and eighty-four (n=384) students were invited to attend this training course, 275 (71.6%) of whom were enrolled in the study. The overall satisfaction rate was 93.8%. The objectives and organisation were 95.6% successful. The quality of the training was 90.3% satisfactory. The choice of topic for the station was satisfactory for 80% of participants, the flow of the session for 86.3%, the quality of the organisation and debriefing for 89%, the interaction between trainers and learners for 90.2%, the motivation of trainers and the reflection stimulated in the learners for 92%. We noted that 93% of students had never participated in training in transfusion safety. Mastery of global skills was total for 67%, partial for 26% and absent for 7%. The students mastered the procedures for checking the identity and grouping of the product to be transfused at 97%, the principle of interpreting the ultimate pre-transfusion test (96%), the purpose of ultimate test at the patient´s bedside 93% with mastery of its technical implementation 83%.

**Conclusion:**

training in transfusion safety was well received, with a satisfactory level of proficiency. This experience can easily be extended to other topics.

## Introduction

Les connaissances des étudiants en médecine en matière de transfusion sont essentielles pour la sécurité de leurs futurs patients [1]. La transfusion sanguine n´est pas sans risque et ses complications font l´objet de préoccupations constantes [1,2]. Malheureusement, la formation médicale au sein des facultés de médecine et des hôpitaux du monde entier abordent peu le sujet de la médecine transfusionnelle et nécessitent un renforcement des programmes universitaires actuels. Dans plusieurs pays, la formation sur la transfusion sanguine n´est toujours pas proposée aux étudiants en médecine pendant leur cursus [2,3]. Les indications transfusionnelles sont plus fréquentes que ce qui est recommandé, ce qui contribue à augmenter les risques liés à cette procédure [4,5]. Les formations pratiques sont essentielles pour garantir la sécurité du processus transfusionnel [6]. Pour pallier à ceci, la faculté de médecine et de pharmacie de Marrakech a dû organiser, à son centre de simulation et d´innovation en science de la santé (CSI2S), des stations formatives en gestes et soins infirmiers, dont la sécurité transfusionnelle, pour les étudiants de troisième année de médecine, afin de renforcer son programme pédagogique. L´objectif de notre étude était d´évaluer cette formation et son impact sur l´amélioration des compétences des étudiants sur la sécurité transfusionnelle.

## Méthodes

**Type d´étude:** il s´agit d´une étude transversale, monocentrique, menée au sein de la faculté de médecine et de pharmacie de Marrakech au Maroc.

**Population cible:** les étudiants de 3^e^ année en médecine, ayant bénéficié de la formation de simulation médicale en « Gestes et Soins Infirmiers », station « Sécurité Transfusionnelle » au titre de l´année universitaire 2020/2021.

**Recueil des données:** cette étude avait porté sur une analyse descriptive longitudinale d´un questionnaire d´appréciation rempli par les étudiants et d´une grille d´évaluation remplie par les médecins formateurs.

**Conception de l´étude:** le programme de la formation « Gestes et Soins Infirmiers » a englobé dans sa réalisation l´ensemble des étudiants de la 3^è^ année en médecine, répartis sur des groupes de 6 personnes. Sur une période de 4 semaines, elle s´est déroulée à une fréquence de 4 groupes par après-midi, 2 groupes en parallèle sur les 2 étages, sessions de rotation sur 6 stations sur une durée de 90 minutes, soit 15 minutes/station/étudiant. Une présentation Powerpoint ainsi qu´une vidéo qui illustre le contrôle ultime pré-transfusionnel ont été présenté pour chaque étudiant. La totalité des étudiants a été informée par une annonce lancée sur le site web de la faculté et aussi sur les réseaux sociaux.

**Analyse des données:** les données ont été collectées à l´aide d´une grille de satisfaction, rempli par les étudiants, délivré à la fin de la séance, composé de trois parties, la première est dédiée aux informations générales et aux données démographiques, la deuxième partie est composée de 11 questions sous forme d´une échelle de « Likert », le codage utilisé était le suivant: a) 1 = Non pas du tout ; b) 2 = Non plutôt pas; c) 3 = Indifférent; d) 4 = Oui plutôt; e) 5 = Oui tout à fait.

La troisième partie du questionnaire comportait des commentaires libres et des questions ouvertes.

Une deuxième grille d´évaluation qualitative de type « maitrisé, partiellement maitrisé, non maitrisé », a été rempli par les formateurs au cours de la séance, afin d´évaluer les compétences acquises pour chaque étudiant. Les formateurs et les personnels responsables du CSI2S ont été avertis au préalable sur le contenu des grilles et les modalités de réponse, afin de diriger toutes les étapes de l´étude dans les meilleures conditions, et d´assurer l´équité d´évaluation et la confidentialité des réponses pour tous les groupes. Le recueil des informations a été réalisé par la même personne, respectant le volontariat et l´anonymat, aucun refus de participation n´a été noté. L´analyse des données a porté sur la statistique descriptive à l´aide du logiciel Excel. Toutes les parties prenantes ont donné leur consentement pour la participation à cette étude.

## Résultats

**Figure 1:**
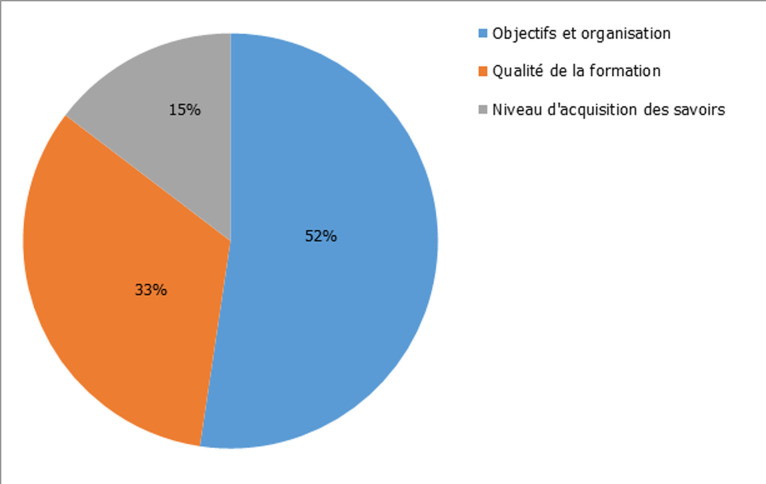
taux de satisfaction global

Deux-cent-soixante-quinze (275) étudiant ont participé à cette étude avec un taux de participation de 71,6%. Chez ces participants, le taux de réponse au questionnaire délivré était de 100%. La majorité des participants était de sexe féminin soit 60% et une sex-ratio H/F de 0,6. La moyenne d´âge était de 20,28 ans avec des extrêmes allant de 19 ans à 21 ans. Le taux de satisfaction globale était de 93,8% (Figure 1). Les objectifs et l´organisation étaient concluants à 95,6%. La qualité de la formation était satisfaisante à 90,3% (Tableau 1). Le choix du thème de la station était satisfaisant à 80%, la fluidité de la séance à 86,3%, la qualité de l´organisation et du débriefing à 89%, l´interaction entre formateurs et apprenants à 90,2%, la motivation des formateurs et la réflexion suscitée chez les apprenants à 92%. Les principales propositions d´amélioration ont été résumées sur le tableau (Tableau 2).

**Tableau 1:** fréquence de satisfaction selon les différents items

	Rubrique	APPRECIATION				Taux de satisfaction
		Très satisfait (A)	Satisfait (B)	Peu satisfait	Pas du tout satisfait	(A+B)
**Objectifs et organisation**	Organisation générale	87,78%	11,85%	0,37%	0,00%	99,6%
	Atteinte des objectifs	87,25%	11,46%	1,29%	0,00%	98,7%
	Temps	69,11%	23,17%	5,41%	2,32%	92,3%
	Locaux	86,19%	13,06%	0,75%	0,00%	99,3%
	Moyens logistiques	86,25%	13,75%	0,00%	0,00%	100%
**Qualité de la formation**	Interactivité formateur-apprenant	91,85%	8,15%	0,00%	0,00%	100%
	Moyens pédagogiques	71,92%	22,31%	3,08%	2,69%	94,2%
	Qualité de la formation	68,25%	13,89%	3,57%	14,29%	82,1%
**Niveau d'acquisition des savoirs**	Maîtrise du contrôle ultime pré-transfusionnel	82,40%	17,60%	0,00%	0,00%	100,0%
**Satisfaction global**		83,03%	13,90%	1,40%	1,67%	96,93%

**Tableau 2:** principales propositions d´amélioration

	Nombre	Pourcentage
**Augmentation du temps de la formation**	55	20%
**Augmentation du nombre de cartes de contrôle ultime**	69	25%
**Généralisation de la formation pour l´ensemble des promotions**	220	80%

**Figure 2:**
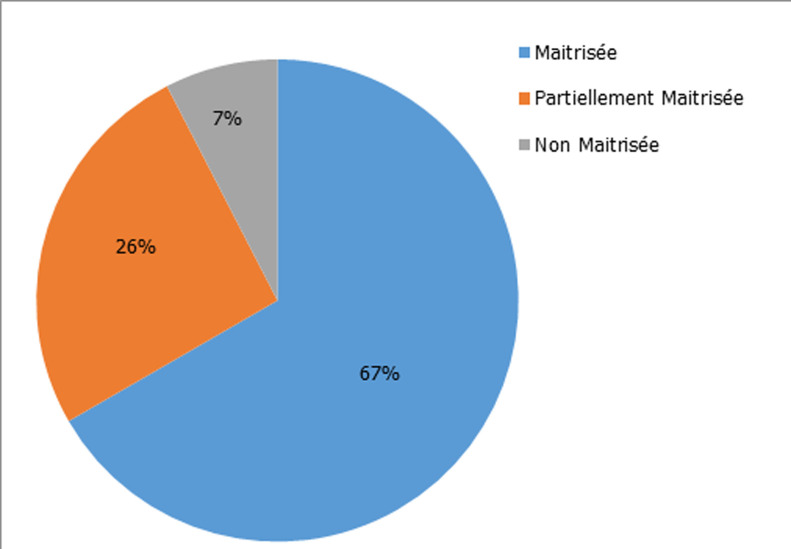
taux de maîtrise globale des compétences

**Figure 3:**
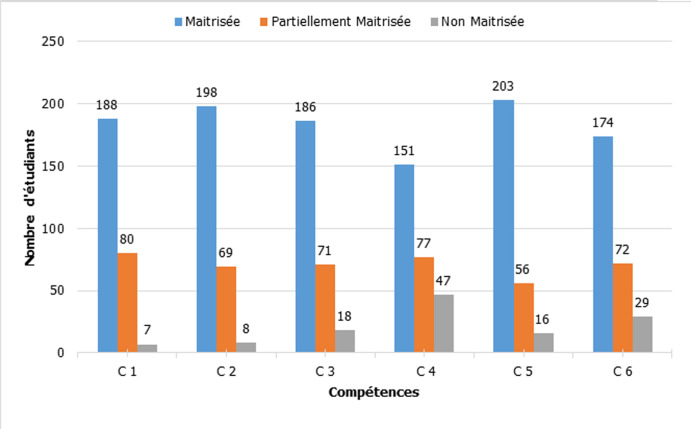
répartition des compétences selon le taux de maitrise

Lors des commentaires libres, 82% des participants ont exprimé leur satisfaction par rapport de la richesse de la station, 90,6% ont apprécié l´initiative et 80% ont demandé une autre séance de simulation. Nous avons noté que 93% des étudiants n´ont jamais participé à une formation sur la sécurité transfusionnelle. Nous avons choisi d´utiliser le taux de maîtrise comme critère d´évaluation des différentes compétences acquises. L´analyse globale des compétences requises a montré une maîtrise totale de 67% et une maîtrise partielle de 26%. Par contre, la non maîtrise de ces compétences a été observée chez 7% des étudiants (Figure 2). L´analyse des résultats (Tableau 3, Figure 3) a montré que 97% des étudiants ont maitrisé les mesures de vérifications de l´identité et du groupage du produit à transfuser, 96% des étudiants ont maitrisé le principe de l´interprétation du contrôle ultime pré transfusionnel et 93% ont maitrisé le but du contrôle ultime au lit du malade. Pour la technique, 83% des enquêtés ont pu réaliser le contrôle ultime de concordance.

**Tableau 3:** évaluation des compétences selon les trois critères de maîtrise

Compétence		Maitrisée	Partiellement maitrisée	Non maitrisée
Les vérifications à faire avant transfusion	**C1**	**68%**	**29%**	**3%**
L´importance de l´utilisation du dispositif de contrôle ultime ABO	**C2**	**72%**	**25%**	**3%**
Le but du contrôle ultime pré-transfusionnel	**C3**	**68%**	**26%**	**7%**
La technique de réalisations d´un contrôle ultime pré-transfusionnel de concordance	**C4**	**55%**	**28%**	**17%**
Le principe de l´interprétation du contrôle ultime de compatibilité	**C5**	**74%**	**20%**	**6%**
Le temps de conservation de la carte de contrôle pré-transfusionnel	**C6**	**63%**	**26%**	**11%**
Compétence globale		**67%**	**26%**	**7%**

## Discussion

Nous avons mené cette étude sur 275 étudiants en médecine. Quatre-vingt-treize pourcent (93%) d´entre eux n´ont jamais reçu de formation préalable sur la sécurité transfusionnelle, ce qui contraste avec de nombreuses autres études [7-9]. Le taux de satisfaction globale était de 93,8% avec atteintes des objectifs organisationnels à 95,6%. La qualité de la formation était satisfaisante à 90,3%, le choix du thème de la station à 80%, la fluidité de la séance à 86,3%, la qualité de l´organisation et du débriefing à 89%, l´interaction entre formateurs et apprenants à 90,2%, la motivation des formateurs et la réflexion suscitée chez les apprenants à 92%. Cette formation mise en place à la faculté de médecine et de pharmacie de Marrakech a reçu un accueil favorable et peut être facilement étendue à d´autres thèmes.

Dans plusieurs pays, tel la France, le Brésil et l´Australie, la formation en sécurité transfusionnelle pour les étudiants en médecine divers d´un centre hospitalier universitaire à un autre. Le temps consacré à ce type de formation est variable, parfois très réduit, voire inexistant. Plusieurs facultés de médecine considèrent cette thématique comme facultative dans leur programme pédagogique [2]. Dans notre étude, sur le plan global, 67% ont maitrisé totalement les compétences prédéfinis. Par contre, 26% ont maîtrisé partiellement ces compétences et 7% ne les ont pas maîtrisés. L´analyse des résultats a montré que 97% des étudiants ont maitrisé les mesures de vérifications de l´identité et du groupage du produit à transfuser, 96% des étudiants ont maitrisé le principe de l´interprétation du contrôle ultime pré transfusionnel, 93% ont maitrisé le but du contrôle ultime au lit du malade avec une maîtrise de la technique à 83%.

Une étude quasi-expérimentale, menée auprès de 190 pédiatres exerçant en Egypte, a montré une amélioration significative du niveau de connaissance de la sécurité transfusionnelle chez les participants après la formation. Seulement 18,4% des participants connaissaient le protocole de dépistage pré transfusionnel, pourcentage qui est passé à 85,8% après la formation. Près de 65,3% ont répondu correctement au quiz sur les réactions transfusionnelles, sans changement significatif après la formation [7]. Le manque de formation en matière de transfusion sanguine dans les programmes médicaux actuels rend primordial la refonte des programmes universitaires [3]. L´utilisation clinique optimale des produits labiles sanguins nécessite des connaissances théoriques et pratiques approfondies en médecine transfusionnelle [4,5]. Etant donné qu´un grand nombre d´étudiants en médecine seront impliqués au cours de leur pratique, qu´elle soit médicale ou chirurgicale, dans des services de soins où la prescription de produits et de dérivés sanguins est nécessaire, il est primordial d´avoir des connaissances appropriées et solides par le biais d´une formation théorique et pratique en sécurité transfusionnelle [6]. Une formation adéquate en médecine transfusionnelle contribue à promouvoir la sécurité du patient et à réduire les erreurs liées à l´acte [1,10,11]. Il est nécessaire donc d´améliorer à la fois les connaissances théoriques et pratiques pour favoriser la bonne pratique transfusionnelle [12]. Tout enseignement supplémentaire pourrait appuyer les études médicales conventionnelles en soulignant les aspects pratiques de la sécurité transfusionnelle [2,13].

## Conclusion

L´originalité de cet enseignement s´appuie sur des points forts comme l´amélioration des compétences des étudiants vis-à-vis de la sécurité transfusionnelle tout en favorisant les échanges entre formateurs et étudiants en nombre limité (maximum de 6 étudiants par groupe). Les points positifs révélés par cette étude étaient le nombre important des participants ainsi que le rôle fondamental de cette formation dans l´amélioration des connaissances des participants sur la médecine transfusionnelle. Les principales limites de l´étude étaient les suivantes: l´échantillon n´était pas représentatif de la population des étudiants en médecine au Maroc, puisque la sélection des participants n´était pas randomisée. L´absence de pré-test afin d´évaluer les niveaux de connaissances avant et après la formation. L´absence d´étude comparable ou similaire dans la littérature. La variété des données et méthodologies utilisées empêche de calculer un effet de taille standardisé et de réalisé une étude comparative.

### Etat des connaissances sur le sujet

- *Le manque de formation en matière de transfusion sanguine dans les programmes pédagogiques actuels;*
- *Le temps consacré à ce type de formation est variable, parfois très réduit, voire inexistant;*
- *Les formations pratiques sont essentielles pour garantir la sécurité du processus transfusionnel*.

### Contribution de notre étude à la connaissance

- *L´importance d´une formation pratique en sécurité transfusionnelle pour les étudiants en médecine en tant que futurs praticiens;*
- *L´amélioration de la maîtrise des objectifs de la sécurité transfusionnelle tout en favorisant les échanges entre formateurs et étudiants en nombre limité;*
- *Cette formation complémentaire aux cours magistraux a reçu un accueil favorable et peut être facilement étendue à d´autres thèmes*.

## Remerciements

Nous remercions le comité d´organisation de la formation au sein de la faculté de médecine et de pharmacie de Marrakech pour l´aide qu´il nous a procuré lors de l´étude. Nous remercions aussi Mr Chakir ABAKARIM pour avoir contribué à la réalisation de l´étude statistique des données.
